# Coronavirus Disease among Persons with Sickle Cell Disease, United States, March 20–May 21, 2020

**DOI:** 10.3201/eid2610.202792

**Published:** 2020-10

**Authors:** Julie A. Panepinto, Amanda Brandow, Lana Mucalo, Fouza Yusuf, Ashima Singh, Bradley Taylor, Katherine Woods, Amanda B. Payne, Georgina Peacock, Laura A. Schieve

**Affiliations:** Medical College of Wisconsin, Milwaukee, Wisconsin, USA (J.A. Panepinto, A. Brandow, L. Mucalo, F. Yusuf, A. Singh, B. Taylor, K. Woods);; Centers for Disease Control and Prevention, Atlanta, USA (A.B. Payne, G. Peacock, L. Schieve)

**Keywords:** sickle cell disease, SCD, hemoglobin, severe acute respiratory syndrome coronavirus 2, SARS-CoV-2, coronavirus, viruses, coronavirus disease, COVID-19, respiratory diseases, zoonoses, United States

## Abstract

Sickle cell disease (SCD) disproportionately affects Black or African American persons in the United States and can cause multisystem organ damage and reduced lifespan. Among 178 persons with SCD in the United States who were reported to an SCD–coronavirus disease case registry, 122 (69%) were hospitalized and 13 (7%) died.

Sickle cell disease (SCD), an inherited hemoglobinopathy that most commonly affects persons of African ancestry, is estimated to affect 1 in 365 Black persons in the United States (*1*). Persons with SCD produce abnormal hemoglobin that causes erythrocytes to become rigid and deform under low oxygen conditions, leading to ischemia–reperfusion injury in the microvasculature with subsequent organ damage and pain. SCD affects nearly every organ system; average life expectancy estimates of affected persons are 43–54 years (*2*,*3*). Persons with SCD are at increased risk for pulmonary disease and pneumonia.

Previous studies have shown that influenza severity and hospitalization rates are higher among persons with SCD than those without SCD (*4*,*5*). Thus, persons with SCD could be at higher risk for development of severe disease if infected with severe acute respiratory syndrome coronavirus 2 (SARS-CoV-2), the causative agent of coronavirus disease (COVID-19). Although empirical data are limited, cases of COVID-19 have been reported in persons with SCD (*6**–**8*), and a study of COVID-19 intensive care unit (ICU) admissions reported 2 (4%) of 48 children had SCD (*9*). We describe a large series of COVID-19 cases and associated deaths among persons with SCD in the United States.

## The Study

The Medical College of Wisconsin established the SECURE-SCD Registry to collect data on COVID-19 cases occurring globally in persons living with SCD. A link to the registry (https://covidsicklecell.org) was distributed to healthcare providers caring for patients with SCD by medical professional and patient advocacy networks and was made available on the Centers for Disease Control and Prevention website. Providers were asked to report all confirmed COVID-19 cases among patients with SCD to this registry; they were specifically asked to report only confirmed COVID-19 cases and to report cases after resolution of acute illness or death. Persons who had the sickle cell trait were not included in this registry, nor were persons with SCD who had suspected but not confirmed COVID-19. Providers were asked to report if the patient died of COVID-19 or complications of COVID-19. All data were deidentified without protected health information.

For each case, providers were asked to complete a short form with questions on demographics; SCD genotype; SCD-related health history; and COVID-19 clinical course, severity, and interventions. In addition to data on COVID-19 clinical severity indicators, such as hospitalization, ICU admission, and death, COVID-19 severity level based on patient manifestations were collected by using established criteria for asymptomatic, mild, moderate, severe, and critical (*10*) (Table).

**Table:** Characteristics of COVID-19 cases and cases resulting in death that were reported to the Secure-SCD Registry, United States, March 20–May 21, 2020*

Characteristic	No. (%) case-patients†	No. (%) deaths†	Case-fatality rate, %
Total	178	13	7.3
Sickle cell disease genotype‡			
HbSS/HbSβ^0^	135 (75.8)	7 (53.9)	5.2
HbSC/HbSβ^+^	42 (23.6)	5 (38.5)	11.9
Mean (SD) age, y	28.6 (14.5)	28.5 (14.6)	NA
Median age, y	26	38	NA
Age range, y	<1–69	12–69	NA
Age group, y			
<19	44 (24.7)	1 (7.7)	2.3
>19	134 (75.3)	12 (92.3)	9.0
Sex			
F	101 (56.7)	6 (46.2)	5.9
M	75 (42.1)	6 (46.2)	8.0
Race/ethnicity			
Black or African American (and not Hispanic or Latino)	142 (79.8)	13 (100.0)	9.2
Hispanic or Latino (and not Black of African American)	21 (11.8)	0	0
SCD health history			
Hospitalized for pain >3 times in past 3 y	96 (53.9)	11 (84.6)	11.5
>1 episodes of acute chest syndrome in past 3 y§	57 (32.0)	3 (23.1)	5.3
Chronic transfusion	23 (12.9)	0	0
Pulmonary hypertension	23 (12.9)	5 (38.5)	21.7
Renal disease			
Albuminuria	27 (15.2)	1 (7.7)	3.7
Decreased renal function	22 (12.4)	3 (23.1)	13.6
SCD nephropathy	17 (9.6)	2 (15.4)	11.8
Stroke			
Overt	22 (12.4)	4 (30.8)	18.2
Silent	10 (5.6)	0	0
COVID-19 case indices			
COVID-19 severity¶			
Asymptomatic	11 (6.2)	0	0
Mild	96 )53.9)	3 (23.1)	3.1
Moderate	32 (18.0)	2 (15.4)	6.3
Severe	30 (16.8)	1 (7.7)	3.3
Critical	9 (5.1)	7 (53.8)	77.8
Accessed care through emergency department	153 (86.0)	13 (100.0)	8.5
Hospitalized	122 (68.5)	11 (84.6)	9.0
Admitted to intensive care unit	19 (10.7)	6 (46.2)	31.6
Ventilator use	10 (5.6)	7 (53.9)	70.0
Received transfusion	68 (38.2)	8 (61.5)	11.8
Received exchange transfusion	16 (9.0)	3 (23.1)	18.6
Received dialysis	4 (2.2)	3 (23.1)	75.0

*Values are no. (%) unless otherwise indicated. COVID-19, coronavirus disease; Hb, hemoglobin; NA, not applicable; SCD, sickle cell disease.  †Numbers do not always add up to total no. case-patients because of missing data. ‡SCD genotypes are HbSS (homozygous for hemoglobin S, a severe phenotype associated with shortest survival); HbSβ^0^-thalassemia (compound heterozygous for hemoglobin S and β^0^-thalassemia, clinically indistinguishable from HbSS); HbSC (heterozygous for hemoglobin S and hemoglobin C, usually moderate clinical severity); HbSβ+-thalassemia (heterozygous for hemoglobin S and reduced amounts of β-globin, usually milder severity). §Acute chest syndrome is a multicausal pneumonia-like illness. ¶COVID-19 severity level classified as asymptomatic, no clinical signs or symptoms during the positive COVID-19 period; mild, symptoms of acute upper respiratory tract infection, including fever, fatigue, myalgia, cough, sore throat, runny nose, and sneezing or gastrointestinal symptoms or digestive symptoms, such as nausea, vomiting, abdominal pain and diarrhea; moderate, pneumonia, with or without clinical symptoms, no hypoxia; severe, early respiratory symptoms or gastrointestinal symptoms followed by dyspnea and hypoxia (O_2_ saturations <92%); critical, acute respiratory distress syndrome, respiratory failure, encephalopathy, shock, coagulopathy, and multiorgan impairment (lung, heart, kidney, brain) that might be life threatening.

This analysis was limited to cases among persons with SCD living in the United States reported during March 20–May 21, 2020. We describe the reported cases and deaths caused by COVID-19 and provide case-fatality rates. Given uncertainty in the sample representativeness and ongoing data reporting to the SECURE-SCD Registry, we view these data as a hypothesis-generating case series. Thus, we did not use statistical tests to assess the significance of differences in mortality rates by subgroup.

As of May 21, 2020, a total of 178 COVID-19 cases were reported to the SECURE-SCD Registry among persons living in 22 states. The mean age of these case-patients was 28.6 years; 57% were female, and 80% were Black (Table). A total of 76% of case-patients had sickle cell types HbSS or HbSβ^0^-thalassemia, which is consistent with estimates of SCD genotype distribution in the United States (*11*). Recent (within the past 3 years) adverse events indicative of vasoocclusive crises were common among case-patients; 54% reported >3 pain episodes requiring hospitalization and 32% reported >1 acute chest syndrome episodes. Prevalence of pulmonary hypertension, previous stroke, renal disease, and use of chronic transfusion therapy were all >10%.

A total of 6% of COVID-19 case-patients were asymptomatic, 54% had mild disease severity, 18% had moderate disease severity, 17% had severe disease severity, and 5% had critical disease severity. Nearly 90% of case-patients accessed care through an emergency department, 69% were hospitalized, 11% were admitted to an ICU, 6% required a ventilator, 38% required a transfusion, and 2% required dialysis.

A total of 13 (7%) patients died (Table). Their mean age was 38.5 years, and >90% were adults. Nearly 40% of deaths were among persons who had genotypes generally associated with milder SCD (types HbSC or HbSβ+-thalassemia). Patients who died had high proportions of frequent pain episodes in the previous 3 years (85%), pulmonary hypertension (39%), decreased renal function (23%), SCD nephropathy (15%), and overt stroke (31%). Of the 13 deaths, 8 were among persons who had severe or critical COVID-19; 5 deaths were observed in persons who had mild or moderate COVID-19.

## Conclusions

Our findings suggest that persons who have SCD and become infected with SARS-CoV-2 have a high risk for a severe disease course and a high case-fatality rate. Among confirmed COVID-19 case-patients reported to the registry, the 69% hospitalization rate, 11% ICU admission rate, and 7% mortality rate are alarming, given that the mean patient age was <40 years. Comparison to a previous report of COVID-19 in the general US population indicates that hospitalization, ICU admission, and case-fatality rates for persons with SCD could be much higher than persons of similar ages in the US population-at-large (*12*). For example, COVID-19 case-fatality rates were reported as <1% for persons 20–44 years of age and for persons 45–54 years of age in the population-at-large (*12*).

These data should be considered in the context that these cases may not be representative of all COVID-19 cases among persons with SCD. There may be bias toward more severe cases in this registry; however, providers were asked to report all COVID-19 cases among their SCD patients. Also, whereas providers were specifically instructed to report only confirmed COVID-19 cases, further guidance was not provided on case confirmation, nor were laboratory testing results included in the registry; thus, we cannot rule out the possibility that a small number of suspected cases were erroneously reported.

Our findings are consistent with expectations based on SCD pathophysiology. SCD can cause multisystem organ damage, life-long disability, and reduced lifespan. Nonetheless, in this case series, COVID-19 deaths occurred in persons who had severe and mild-to-moderate SCD genotypes. Also, deaths occurred in COVID-19 case-patients classified as having mild-to-moderate disease severity. We did not have data to assess whether this finding was caused in part by an impact of SARS-CoV-2 exacerbating preexisting cardiac or SCD concurrent conditions; further study is needed.

Persons with SCD face socioeconomic and healthcare access disparities that might compound their already high risk for severe COVID-19 because of their underlying disease and concurrent conditions. SCD complications might negatively impact educational achievement and employment (*13**,**14*). Accessing appropriate healthcare is difficult given the lack of providers with SCD expertise. SCD patients might delay seeking care, and emergency department visits are high among this population that is placed at increased risk for poor health (*14*).

Our findings underscore the need to consider the unique circumstances faced by high-risk subgroups. SCD is one of many possible explanations for higher rates of illness and mortality from COVID-19 among Black populations in the United States. As with all high-risk groups, staying home, social distancing, and hand hygiene are necessary for persons with SCD (*15*), along with prompt care-seeking if COVID-19 is suspected or SCD complications arise. In addition, specific socioeconomic and healthcare access challenges that many persons with SCD face (i.e., social determinants of health) need to be considered in implementing prevention measures.
